# Examining the Impact of COVID-19 Lockdown in Wuhan and Lombardy: A Psycholinguistic Analysis on Weibo and Twitter

**DOI:** 10.3390/ijerph17124552

**Published:** 2020-06-24

**Authors:** Yue Su, Jia Xue, Xiaoqian Liu, Peijing Wu, Junxiang Chen, Chen Chen, Tianli Liu, Weigang Gong, Tingshao Zhu

**Affiliations:** 1 CAS Key Laboratory of Behavioral Science, Institute of Psychology, Chinese Academy of Sciences (CAS), Beijing 100101, China; suy@psych.ac.cn (Y.S.); wupeijing@bupt.edu.cn (P.W.); tszhu@psych.ac.cn (T.Z.); 2 Department of Psychology, University of Chinese Academy of Sciences, Beijing 100049, China; 3 Factor-Inwentash Faculty of Social Work& Faculty of Information, University of Toronto, Toronto, ON M5S 1A1, Canada; 4 School of Computing, Beijing University of Posts and Telecommunications, Beijing 100876, China; 5 School of Medicine, University of Pittsburgh, Pittsburgh, PA 15260, USA; Juc91@pitt.edu; 6 Middleware System Research Group, University of Toronto, Toronto, ON M5S 1A1, Canada; Chenchen@eecg.toronto.edu; 7 Institute of Population, Peking University, Beijing 100871, China; tianli.liu@pku.edu.cn; 8 School of Sociology, Wuhan University, Wuhan 430072, China; gongweigangruc@163.com

**Keywords:** impact of COVID-19 lockdown, public health emergencies, psycholinguistic analysis, psychological states

## Abstract

Many countries are taking strict quarantine policies to prevent the rapid spread of COVID-19 (Corona Virus Disease 2019) around the world, such as city lockdown. Cities in China and Italy were locked down in the early stage of the pandemic. The present study aims to examine and compare the impact of COVID-19 lockdown on individuals’ psychological states in China and Italy. We achieved the aim by (1) sampling Weibo users (geo-location = Wuhan, China) and Twitter users (geo-location = Lombardy, Italy); (2) fetching all the users’ published posts two weeks before and after the lockdown in each region (e.g., the lockdown date of Wuhan was 23 January 2020); (3) extracting the psycholinguistic features of these posts using the Simplified Chinese and Italian version of Language Inquiry and Word Count (LIWC) dictionary; and (4) conducting Wilcoxon tests to examine the changes in the psycholinguistic characteristics of the posts before and after the lockdown in Wuhan and Lombardy, respectively. Results showed that individuals focused more on “home”, and expressed a higher level of cognitive process after a lockdown in both Wuhan and Lombardy. Meanwhile, the level of stress decreased, and the attention to leisure increased in Lombardy after the lockdown. The attention to group, religion, and emotions became more prevalent in Wuhan after the lockdown. Findings provide decision-makers timely evidence on public reactions and the impacts on psychological states in the COVID-19 context, and have implications for evidence-based mental health interventions in two countries.

## 1. Introduction

The COVID-19 (Corona Virus Disease 2019) pandemic is a global health emergency that is having a profound impact on the physical and mental health of people [1,2,3]. Many countries have taken strict quarantine measures as an intervention: cities locked down, school closure, mass gathering ban, public event prohibition, and self-isolation. A study conducted in China shows that lockdown has been effective in postponing the spread of COVID-19 [4]. However, strict quarantine interventions may have negative impacts on mental health [5,6]. It is essential to investigate the psychological effects of the lockdown which could make an influence on the execution of measures on epidemic containment. Studies find that public reactions to SARS (Severe Acute Respiratory Syndrome) in 2003 and the Ebola virus disease in 2014 have impeded infection control to an extent [7,8]. Moreover, quarantine measures are making psychosocial impact on individuals more severe [9]. This study intends to explore how the lockdown affects the psychological states.

The “first case” of COVID-19 was identified in Wuhan [10], which was the epicenter of the coronavirus outbreak in China. To stop the spread of COVID-19, China declared the lockdown of Wuhan on 23 January 2020, which was the first city placed on lockdown during this pandemic in China and affected over 11 million people [11]. In Europe, Italy was the first country facing the pandemic [12] and taking actions (e.g., banned flights) [13]. Lombardy in Italy was the most affected area by COVID-19 [14]. On 8 March 2020, the Italian government announced a quarantine zone that covered most of northern Italy, including Lombardy. Lombardy had a population of over 10 million, which was comparable with Wuhan. This quarantine measure was considered as the most aggressive response taken in any region beyond China [15]. Taken all these together, we chose Wuhan and Lombardy as research regions to investigate the impacts of the lockdown.

Recent studies used the self-report questionnaire approach to examine the psychological responses during the lockdown in different countries, including Italy, India, and China [12,16,17]. However, these studies rely on retrospective and time-lagged surveys and interviews. These approaches have limitations in accessing psychological statuses before the lockdown. That is, there is recall bias inevitably when people are required to recall a past period in the retrospective study.

Social media plays a vital role in recording the reactions, opinions, and mental health features of social media users [18] Previous studies suggest that the language use and psychosocial expressions on social media data provide indicators of mental health [19,20,21]. In China, Sina Weibo is the leading social media service provider. Upon the end of 2019, the number of daily active users of Sina Weibo reached 222 million. Twitter is one of the most-used social media platforms in Italy. Weibo and Twitter provide vast amounts of user’s online behavioral records for researchers. Although there are some differences between Weibo and Twitter when comparing the functions and other features of platforms, they both serve as the online environment of expression and communication, providing us features of contents related to this study. Thus, we collected Chinese social media data from Weibo and used Twitter to acquire Italian social media data.

Existing studies have widely used the Language Inquiry and Word Count (LIWC) and confirmed it as a valid tool for psychometric analysis [22]. The LIWC dictionary has multiple versions of different languages, including English [23], French [24], Italian [25], and Dutch [26]. The LIWC dictionary includes many word categories of linguistic features that are related to mental processes and human behaviors [22]. For example, the word category of personal pronouns reflects attentional allocation [22].

In this study, we used the simplified Chinese version of LIWC and Italian LIWC to measure people’s psychological status before and after the lockdown in Wuhan and Lombardy, respectively. By using psycholinguistic analysis, we aim to identify the psychological effects of the lockdown on individuals in Wuhan and Lombardy.

## 2. Materials and Methods

We downloaded active user’s posts from Weibo and Twitter as our dataset. The research protocol was approved in advance by the Ethics Committee of the Institute of Psychology, Chinese Academy of Sciences (approved number: H15009).

We extracted the linguistic features using the Simplified Chinese LIWC dictionary (SCLIWC) [27] and the Italian LIWC dictionary [25]. Given both SCLIWC and Italian LIWC share LIWC dictionary structure, there are many common words in SCLIWC and LIWC. To make the result of Wuhan and Lombardy comparable, we only analyzed the common word categories between SCLIWC and Italian LIWC. The selection procedure of common categories is as follows:

1. A native Italian speaker who is fluent in English translated the names of Italian LIWC word categories into English.
2. We translated the Chinese names of SCLIWC word categories into English.
3. We selected the common names between two translation versions. As for the names sharing similar meanings, such as “tentative” from SCLIWC and “possibility” from Italian LIWC, we checked the meaning of words belonging to this word category in Italian LIWC and SCLIWC to determine whether the two names represented the same kind of word category.

### 2.1. Dictionary Processing

Some word categories are unique in SCLIWC or Italian LIWC. By comparing, there are 26 word categories only existing in SCLIWC, such as *quantity unit, interjunction*, and *tense mark words*. In Italian, people conjugate verbs when they follow different subjects. Moreover, people can infer the subject of the sentence from verb conjugation. As the subject in a sentence is dropped sometimes, conjugations (*I_verb, We_verb, You_verb, You_plural_verb, HeShe_verb, They_verb*) can reveal the use of pronouns more accurately compared to pronouns (*I, We, You, You plural, HeShe, Them*). Thus, we regard the use of conjugations, the same as pronouns in our study. Additionally, we found that 28 word categories only exist in Italian LIWC. In this study, we kept the common word categories in both SCLIWC and Italian LIWC and got 51 common word categories for further analysis.

### 2.2. Weibo Data

The Chinese samples are from the Weibo data pool containing 1.16 million active Weibo users [28]. In this study, we selected active Weibo users from the data pool by the following criteria:

1. Published at least one original post on average each day from 9 January 2020 to 5 February 2020 (i.e., two weeks before and after the lockdown);
2. Individual users only, excluding any organizations;
3. Locate at “Wuhan, Hubei” by the geo-location in the user profile.

We finally got 850 Weibo users and downloaded their posts published from 9 January 2020 to 5 February 2020. For each Weibo user, we divided the posts into two groups. For example, all posts published before the date 23 January 2020, are labeled as “before lockdown” group. In contrast, those posts published after the data 23 January 2020 (23 January included) are labeled as “after lockdown” group. We employed the TextMind system to extract linguistic features [29] in each of the two groups for all sampled Weibo users. Then, we used the LIWC dictionary containing 51 common word categories to extract psycholinguistic features and calculated word frequencies of each word category in the dictionary. The final dataset included the word frequencies of two groups from 850 Weibo users.

### 2.3. Twitter Data

We sampled Italian Twitter users’ messages as our Twitter data. We downloaded Tweets of users whose location authentication is Lombardia, Italy. There are 3,650,380 tweets acquired. We then selected Italian Twitter users as follows:

1. Published at least one original tweet (not retweet) from 23 February 2020 to 21 March 2020 (that is, two weeks before and after the lockdown);
2. All tweets in Italian only.

We acquired 14,269 Tweets from 188 unique Twitter users. We divided these Tweets into two groups as well. We gathered each user’s tweets and labeled the Tweets posted before 8 March 2020, as “before lockdown” Tweets and Tweets posted after 8 March 2020 (March 8 included) as “after lockdown” Tweets, respectively. We filtered out the users if only emoji, numbers, web links, “@” and “#” were published in either “before lockdown” or “after lockdown” tweets. We finally acquired 120 Twitter users. Then, we extracted every user’s linguistic features from “before” and “after” tweets by using the same dictionary used in Weibo data and calculated word frequencies of each word category.

We conducted Wilcoxon tests to examine the differences between linguistic characteristics before and after the lockdown. SPSS (Statistical Product and Service Solutions) 26.0 (International Business Machines Corporation, Armonk, NY, USA). Released 2019. IBM SPSS Statistics for Macintosh, Version 26.0. was used during data analysis, which was published by IBM (International Business Machines Corporation, Armonk, NY, USA).

## 3. Results

### 3.1. Wuhan Weibo Users

In this study, we compared the word frequencies of 51 LIWC categories before lockdown with after lockdown in Wuhan. Results showed that the frequencies of 39 word categories were statistically significantly different before and after Wuhan lockdown. We identified 16 out of 39 significant categories with absolute values of effect size greater than 0.2, including function words (e.g., *I, we*), relative words (*motion, time*), personal concerns words (*home, money, religion*), affective process words (*negative emotion, affect*), social words (*humans, social*), and cognitive mechanism words (e.g., certain, inhibition). As shown in Table 1, the *first-person plural pronoun* is of high effect size (*p* < 0.001, effect size d = 0.674), which means users used more words of the *first-person plural pronoun* significantly after the lockdown. In addition, Weibo users mentioned more in *religion*, *social*, *negative emotion*, *home*, *affect* after Wuhan lockdown. Meanwhile, we found significant decreases in the frequencies of some word categories, such as *motion, I, money,* and *time* after the lockdown.

### 3.2. Lombardy Twitter Users

We compared the word frequencies of the 51 LIWC categories before and after Lombardy lockdown (8 March 2020). Results showed that the frequencies of 10-word categories were significantly changed. Among them, the number of word categories with absolute values of effect size greater than 0.2 is five-word categories, including personal concerns words (*leisure, home*), affective process words (*anxiety*), and cognitive mechanism words (*discrepancy, possibility*). As shown in Table 2, there are increases in the frequencies of *discrepancy, home, leisure*, and *possibility*. Meanwhile, we observed significant decrease in the frequency of *anxiety* after the lockdown.

### 3.3. Comparison between Wuhan and Lombardy

We presented the word categories whose frequencies significantly changed after the lockdown both in Wuhan and Lombardy in Table 3, including *home* and *discrepancy*. In both Wuhan and Lombardy, the frequencies of *home* and *discrepancy* words increased after a lockdown.

## 4. Discussion

The present study uses the Chinese version of LIWC and Italian version of LIWC to extract the psycholinguistic features from social media users’ posts. Examinations of the features allow us to access the changes of psychological status before and after the lockdown in Wuhan and Lombardy.

### 4.1. Similarities between Wuhan and Lombardy

The frequencies of some word categories increase in both Wuhan and Lombardy after the lockdown, including *discrepancy* and *home* words. These linguistic features imply that social media users’ psychological states were impacted after the COVID-19 lockdown, in both Wuhan and Lombardy.

The increased use of *home* words is related to mobility control after the lockdown in Wuhan and Lombardy. Researchers estimated that mobility and social contacts in China during the lockdown dropped about 80%, concerning a baseline set on 1 January 2020 [30]. Moreover, Google reported a 23% increase in residential location activity in Lombardy during lockdown compared to baseline [31]. These reports indicate people spend more time at home and spend less time outdoors during a lockdown, which is consistent with more use of *home* words.

The frequency of *discrepancy* words increases after the lockdown in Wuhan and Lombardy. Besides, we observe the increased use of *inhibition* and *certain* words after the lockdown in Wuhan. Previous study suggests that the uses of *discrepancy, Inhibition,* and *certain* words reflect the change of degree of cognitive processing [32]. Furthermore, cognitive processing indicates that individuals make efforts to make sense of the environment [32]. Residents in Wuhan and Lombardy attempt to figure out what has happened after the lockdown. Thus, they could adjust their attitudes and lifestyles to accommodate new circumstances during the COVID-19 pandemic.

### 4.2. Differences between Wuhan and Lombardy

We observe there are some differences between Wuhan and Lombardy after a lockdown in the use of LIWC word categories.

We find significant changes in three-word categories in Lombardy, including *tentative*, *anxiety*, and *leisure* words. The use of *tentative* words increases after a lockdown in Lombardy. The previous study shows that people may use *tentative* language (e.g., maybe, perhaps, guess) when they feel uncertain or insecure about their topic [22]. Our findings suggest that people tend to use *tentative* words during the lockdown. Losing direct social contacts during the lockdown contributes to make residents feel losses of recreation, freedoms, and supports [1]. Such a sense of loss means losing control of their healthy life, and people are likely to feel uncertain about the upcoming situation. Tweets reveal that people in Lombardy express such feelings on social media. However, we do not observe such change in Wuhan, suggesting that people in Wuhan do not convey the emotions of uncertainty in their posts on Weibo.

Our results show that Twitter users in Lombardy use more *leisure* words in their posts after the lockdown. The increased use of *leisure* words implies more focus on leisure activities after a lockdown in Lombardy. According to the news reports from CNBC (Consumer News and Business Channel), Italians turn to music to boost morale during lockdown [33], which might be expressed in the use of *leisure* in Tweets. On the contrary, we do not find the same change in the use of *leisure* words in Wuhan. With the rapid growth of the pandemic, some people might focus more attention on the latest news of this disease on Weibo and discuss less about leisure after the lockdown. Moreover, some people may talk more about leisure and recreation after the lockdown, considering that the Lunar New Year holiday was in the lockdown period (25 January 2020, is the Spring Festival in China). Considering these two facets, we may find it reasonable to observe no change in the use of *leisure* words in Wuhan.

The use of *anxiety* words decreases in Lombardy. *Anxiety* reveals self-reported stress [34]. Our results imply that people feel less stressed after Lombardy lockdown. However, people do not experience any change of stress in Wuhan. Researchers find that unrealistic optimism is more evident for European North Americans [13], which might be related to the different responses in the level of stress between Lombardy and Wuhan after the lockdown. However, our results are not consistent with existing studies [12,35]. Rossi and colleagues consider that the strict measures of the lockdown in Italy serve as an unprecedented stressful event [12]. Besides, Ahmed and colleagues find that 29% of respondents report different levels of anxiety related to lockdown at home in China [35]. Such differences could be due to different research methods, design, measurements, and timeframe used in the study.

Some word categories are changing significantly after the lockdown only in Wuhan. The uses of *first-person plural pronouns, second-person plural pronouns, religion, social, negative emotions, humans, certainty, affect, inhibition,* and *prepositions* words increase. In contrast, the uses of *motion, first-person singular pronouns, time,* and *money* words decrease after the lockdown in Wuhan.

In Wuhan, the uses of the *first-person plural pronouns, second-person plural pronoun* increase after a lockdown, while the use of *first-person singular pronoun* decreases. Previous reports confirm that *first-person singular pronouns* show attention to the self, whereas most other pronouns suggest attention to other individuals [36]. Moreover, “we” implies a sense of group identity sometimes [37]. Results suggest that people switch their attention from themselves to others and the communities after the lockdown. Besides, the increased use of “we” indicates that people focus more on the group, become more united, and share more group identity after a lockdown, which is consistent with some researchers’ opinions [1]. China has a collectivistic culture, and Italy has an individualistic culture [38]. Results show that the increased use of other pronouns and decreased use of *first-person singular pronouns* suggest a collectivistic culture in China. At the same time, the absence of such a consequence in Lombardy might be related to the individualist culture. Researchers find that people sharing collectivist values stress more communal coping as a resource to cope with collective traumatic events [39], which is consistent with our research conducted in the context of the lockdown.

Holmes and colleagues find that higher levels of the use of emotion words indicate more immersion in the negative event [40]. In the study, we find that a higher degree of immersion [22] evidenced by the frequent use of emotion words (*negative emotion* and *affective process* words). Therefore, people in Wuhan might get more emotional and are at a deeper level of immersion in negative emotions after the lockdown. However, we do not observe such a situation in Lombardy.

Besides, we also find a decrease in the use of *motion* words after a lockdown in Wuhan. Our results are consistent with the previous mobility study of Wuhan [41], suggesting that stringent mobility control leads to the reduction of movement in Wuhan. Google’s location mobility report in Lombardy shows an 85% decrease in activities at transit stations, a 57% drop in activities at workplaces, an 86% drop in activities at parks, and a 94% drop in activities at retail and recreation from 15 March 2020 to 26 April 2020. However, our results do not identify a significant change of mobility in the use of *motion* words in Lombardy.

The increased use of *social* words in Wuhan after the lockdown suggests the focus on social concerns and social support [22]. Social support can make people feel better about their situation and reinforce the belief that they have access to support resources [16,42]. Thus, seeking social support is considered adaptive for people during a lockdown. In contrast, we do not observe such a change in Lombardy.

Table 1 shows increases in the uses of *religion* and *humans* words, while decreases in the uses of *money* and *time* words after Wuhan lockdown. Content word categories explicitly reveal where individuals are focusing, including death, sex, and money [22]. Moreover, our results suggest people focus more on humans and religion, while less on money and time during the lockdown. The previous study finds that religion can bring more positive and comforting emotions, and people tend to use it when suffering from emergencies such as stress or death [43]. The increases in the use of *religion* words suggest an adaptive behavior during the lockdown. Moreover, the decreased use of *money* words may relate to fewer transactions under strict restrictions. In contrast, we do not identify any changes in these word categories among Lombardy Tweets. This result in Lombardy Tweets suggests that residents in Lombardy do not change their focus level on religion, human, time, and money after lockdown.

Besides, we find an increase in the use of *prepositions*. Previous research shows that *prepositions* signal more complex expression and detailed information about a topic [44]. The increased use of *prepositions* in Wuhan indicates broader and more in-depth discussions that occurred on Weibo after lockdown. However, such a change is not identified in Lombardy.

Study findings have implications for decision-makers, public health authorities, and practitioners. First, considering the efforts of adjusting to the changing environment in both Wuhan and Lombardy after the lockdown, decision-makers should ensure the supply chain functions as usual to ensure people’s confidence in having the control of their lives. Besides, public health authorities and practitioners could adjust their focus of service given the changes in residents’ attention after lockdown. For example, people in Wuhan expressed more stress and negative emotions, public health authorities and practitioners should take interventions to comfort them and relieve stress, such as the online consulting service and indoor activities. Notably, the support for individuals with pre-existing mental or physical health issues is also needed. Meanwhile, people did not show significant stress in Lombardy. Public health communities and practitioners might focus more on the popularization of pandemic prevention knowledge and the reinforcement of protection awareness.

There are several limitations. First, our samples were from selected active social media users only. The results have a limitation in generalizing to the whole population. Second, language differences exist between Chinese and Italian. While processing Italian text, some inevitable errors may occur because of the apostrophe. Third, we do not have access to the users’ IP, and location authentication is self-reported. There are some studies also applying self-reported location authentication to identify users’ locations [45]. Fourth, the bias existing in two different platforms possibly influences the results of our study. Twitter users generally use more hashtags than Weibo users, which shows that Twitter users seem to be more eager to publicize their posts [46]. In addition, Weibo users have a stronger tendency to post positive content compared to Twitter users [46]. Considering these differences between Twitter and Weibo, future studies should find methods to deal with these differences to avoid biases when employing data from Weibo and Twitter.

## 5. Conclusions

This study examined the changes in psycholinguistic features before and after a lockdown in Wuhan and Lombardy. We compared the differences in frequencies of LIWC word categories before and after lockdown and found that the number of word categories whose frequencies were significantly changed is more in Wuhan than in Lombardy. We found significant changes in the use of function words, relative words, personal concerns words, affective process words, social words, and cognitive mechanism words among Wuhan users’ posts. We also found significant changes in the frequencies of personal concerns words, affective process words, and cognitive mechanism words in Lombardy. Individuals focus more on home and express more levels of the cognitive process after a lockdown in both Wuhan and Lombardy. In Lombardy, the level of stress decreases, the use of *leisure* increases. In Wuhan, people convey more emotion expressions, more feelings of uncertainty, and more focus on groups after the lockdown. Results inform decision-makers, public health authorities, and practitioners the potentially different impacts of city lockdown on individuals in the two countries, and contribute to the cultural-based psychological responses.

## Figures and Tables

**Table 1:** Word categories with significant differences between “before” and “after” in Weibo (*n* = 850).

SCLIWC	Category Name	Before Lockdown		After Lockdown		*p*	Effect Size d
		M1	SD1	M2	SD2		
We	First-person plural Pronoun	0.00116752	0.001512455	0.002442816	0.002243907	0.000 ***	0.674
Motion	Motion	0.025831994	0.019530379	0.018399454	0.009675841	0.000 ***	0.455
Religion	Religion	0.002540956	0.002514763	0.003610935	0.002649443	0.000 ***	0.401
I	First-person singular pronoun	0.012875111	0.007807225	0.010321953	0.006449756	0.000 ***	0.391
Social	Social	0.029713574	0.012688112	0.034711722	0.012327484	0.000 ***	0.375
Youpl	Second-person plural pronoun	0.000306648	0.000688167	0.000724517	0.001041388	0.000 ***	0.364
Negemo	Negative emotion	0.007779827	0.005568892	0.009515898	0.004745883	0.009	0.334
Time	Time	0.027584026	0.011749097	0.02408661	0.009783141	0.000 ***	0.325
Certain	Certain	0.006946484	0.004624027	0.00833264	0.003605291	0.000 ***	0.308
Home	Home	0.002152596	0.002465285	0.002935781	0.002730609	0.000 ***	0.306
Humans	Humans	0.008192476	0.005123264	0.010069752	0.004594877	0.000 ***	0.306
Money	Money	0.007305661	0.007514546	0.005138734	0.004664296	0.000 ***	0.278
Preps	Preposition	0.015862793	0.008304327	0.017603575	0.006868765	0.000 ***	0.247
Discrep	Discrepancy	0.011271717	0.005658052	0.012859929	0.005581719	0.000 ***	0.235
Inhibition	Inhibition	0.002031626	0.002027422	0.002571519	0.001937226	0.000 ***	0.234
Affect	Affect	0.03540768	0.012714117	0.039041458	0.013830077	0.000 ***	0.230

*** *p* < 0.001. SCLIWC—the Simplified Chinese LIWC (Language Inquiry and Word Count) dictionary; M1—the mean of the “before lockdown” group; SD1—the standard deviation of the “before lockdown” group; M2—the mean of the “after lockdown” group; SD2—the standard deviation of the “after lockdown” group.

**Table 2:** Words with significant changes between “before lockdown” and “after lockdown” in Lombardy (*n* = 120).

Italian LIWC Category (in Italy)	English Translation	Before Lockdown		After Lockdown		*p*	Effect Size d
		M1	SD1	M2	SD2		
Discrep (Discrepanza)	Discrepancy	0.009287369	0.012033078	0.013716728	0.017095823	0.001	0.271
Ansia	Anxiety	0.002470567	0.005926371	0.000921978	0.002132726	0.002	0.245
Casa	Home	0.002778005	0.005026976	0.005169261	0.008826728	0.001	0.233
Possib (Possibilità)	Possibility (tentative)	0.008970828	0.010353838	0.012373076	0.017164373	0.009	0.210
Svago	Leisure	0.004710796	0.006753619	0.007065489	0.009775871	0.017	0.207

LIWC—Language Inquiry and Word Count; M1—the mean of the “before lockdown” group; SD1—the standard deviation of the “before lockdown” group; M2—the mean of the “after lockdown” group; SD2—the standard deviation of the “after lockdown” group.

**Table 3:** Words with significant changes in both Wuhan and Lombardy.

Wuhan				Lombardy			
SWLIWC	English Name	*p*	Effect Size d	Italian LIWC	English Name	*p*	Effect Size d
Discrep	Discrepancy	0.000 ***	0.235	Discrep	Discrepancy	0.001	0.271
Home	Home	0.000 ***	0.306	Casa	Home	0.001	0.233

*** *p* < 0.001.
